# Diurnal variations of psychometric indicators in Twitter content

**DOI:** 10.1371/journal.pone.0197002

**Published:** 2018-06-20

**Authors:** Fabon Dzogang, Stafford Lightman, Nello Cristianini

**Affiliations:** 1 Intelligent Systems Laboratory, University of Bristol, Bristol, United Kingdom; 2 Henry Wellcome Laboratories for Integrative Neuroscience and Endocrinology, University of Bristol, Bristol, United Kingdom; University of Lübeck, GERMANY

## Abstract

The psychological state of a person is characterised by cognitive and emotional variables which can be inferred by psychometric methods. Using the word lists from the Linguistic Inquiry and Word Count, designed to infer a range of psychological states from the word usage of a person, we studied temporal changes in the average expression of psychological traits in the general population. We sampled the contents of Twitter in the United Kingdom at hourly intervals for a period of four years, revealing a strong diurnal rhythm in most of the psychometric variables, and finding that two independent factors can explain 85% of the variance across their 24-h profiles. The first has peak expression time starting at 5am/6am, it correlates with measures of analytical thinking, with the language of drive (e.g power, and achievement), and personal concerns. It is anticorrelated with the language of negative affect and social concerns. The second factor has peak expression time starting at 3am/4am, it correlates with the language of existential concerns, and anticorrelates with expression of positive emotions. Overall, we see strong evidence that our language changes dramatically between night and day, reflecting changes in our concerns and underlying cognitive and emotional processes. These shifts occur at times associated with major changes in neural activity and hormonal levels.

## Introduction

Our internal states are characterised by multiple dimensions of psychological processes, of which two -emotional and cognitive processing- are of major importance for the maintenance of good mental health. Their expression is both individual and situation dependent and results in observable states or behaviours [1], such as anger, hesitancy or sadness. In everyday life our internal status interacts with our current concerns, so one may turn to causal thinking to cope with work or become positively affected when attending to family matters. Emotional and cognitive processing is also dependent on the stage of the sleep/wake cycle, which modifies the activity of circuits including the brain stem alerting pathways including the midbrain raphe. Indeed, it is clear that circadian genes modify key circuits which impact on mood and reward circuitry [2]. In addition to neural circuits, circulating hormones such as cortisol contribute to optimal neural function and the maintenance of good mental health.

Observing temporal variations in the expression of our internal state requires the investigation of large populations of individuals sampled at an adequate rate which has previously proven difficult [3]. Previous attempts have relied on subjective reporting with inherent problems of self-reporting and recall bias, they have also been limited in their size, or in the duration of the period of observation. In the present study, we leverage the measure of a range of psychometric variables sampled in an uncontrolled population of anonymous individuals to infer the average situation or state in which those persons are at different times of their daily routines. Twitter content was sampled hourly in a four years interval across the whole United Kingdom: we have monitored the usage of cognitive and emotional words from the psychometric word lists defined in the 2015 version of Linguistic Inquiry and Word Count (LIWC) [4]. We have also computed a number of temporal indicators relative to everyday life concerns as well as numerous standard linguistic properties describing the writing style amongst other properties. Our temporal indicators of cognitive and emotional activity, formed in a robust way, capture periodic variations in the collective psychology of the general population of UK Twitter users.

Psychometric patterns in language have been repeatedly found in various settings. Measuring psychometric variables from the content of admission essays written by US students, previous works have related the writing style to academic performance, suggesting a bias towards categorical thinking in the ways academics define success [5]. Using a similar approach, poets who committed suicide have been found to differ in their writings from poets who died a different death [6]. Text-based learning models have also revealed consistent emotional constructs in the content of anonymous suicide notes [7]. The present study relies on a total of 73 psychometric variables, and seeks to identify the temporal mechanisms behind our emotional and cognitive activity. While the cognitive processes refer to the ways people think (e.g causal, categorical or dynamical thinking), the emotional processes refer to the ways people feel (e.g. positive or negative emotion).

The specific content generated on the social media has been studied to observe temporal variations in the collective expression of our emotional processes [3,8,9,10]. These processes show a complex hierarchy of positively and negatively valenced emotions. Finer distinctions separate them further: the emotion of anger has been shown to follow a stable pattern in the 24-h day at different points of the week and year, while the emotion of sadness has been found to vary in response to these changing environmental conditions, with higher levels of interaction observed with the onset of sunlight exposure [3]. Positive and negative states have been found to remain independent in various settings [11,3,8] suggesting different mechanisms behind their expression. In a previous study we identified two distinct patterns of emotional variations within the circadian rhythm, with the positive emotions showing a bi-phasic behaviour, and the emotion of anger showing variations that inversely mirror the variations of plasma cortisol concentrations peaking early in the morning and slowly decreasing for the rest of the day [3].

The temporal indicators we analyse reflect the changes that occurred in the contents of millions of anonymous tweets. We conduct a systematic analysis of their periodicity, and reveal the main factors behind their diurnal variation. Altogether, our results indicate two independent patterns of thinking expressed over the 24-h day. Categorical thinking is dominant in the early morning. During the weekdays, this time corresponds with the morning rush hour, marked with low mood. In contrast at the weekend this becomes a feel-good time. Existential thinking is dominant late in the night, during this time the positive emotions are low, *death* and *religion* are the main concerns in the population.

## Materials and methods

### Data

Twitter contents from the 54 largest cities in the United Kingdom were sampled every hour using the Twitter search API, without specifying keywords or hashtags, and complying with Twitter’s Terms of Service (https://twitter.com/en/tos). For each tweet, we collected the anonymised textual content, a collection date and time, and information about the location of the tweet (within 10km of one of the 54 urban centres). We automatically removed messages containing standard holiday greetings as they contained mood-related words while not necessarily representing an expression of mood (see Materials and methods - Greeting messages). Due to issues in the collection we removed the year 2012 and the months of November and December 2014 from our analyses. As a result, we obtained 800M individual tweets and 7B word occurrences covering the time intervals between January 2010 and November 2014. Each tweet was tokenized using a tool designed specifically for Twitter text [12]. Hyperlinks, mentions and hashtags were discarded, along with punctuation and words containing only special characters (e.g. emoticons). A summary of the dataset is provided in Table 1. The time series of the raw frequency of each word necessary to reproduce this study is publicly available (https://doi.org/10.5061/dryad.f61v3tj).

**Table 1:** Summary of data and sampling.

sampling interval	4 years
sampling frequency	hourly
sampling locations	54 largest urban centers in the UK
sampling start	January 2010
sampling end	November 2014
sampling issues	year 2012 was removed
population size	N/A
Volume	800M tweets and > 7B word occurrences

Anonymising the data at time of collection implies that we cannot distinguish individuals or account for the likely changes in population that occur between night and day. This is required by our user privacy policy, also dominant chronotypes may emphasize some of the properties exposed in our analyses as we cannot separate the variation due to population changes or due to changes in the same individuals. In the uncontrolled population under analysis we expect each anonymous sample of text collected, following the same procedure across days and months, to be representative of the collective content generated in the United Kingdom. We cannot rule out the presence of data posted by bots accounts, but we control for this risk in various ways. First, we verified by hand a sample of data, estimating that tweets generated to promote links to news stories were less than 1.5% at each given hour of the day. Then we observe that in order to affect our results, bots would need to be geolocated in all or most of the 54 largest UK cities, remain active over the 4 years of this study, and present the same diurnal pattern in their content. We believe that the design of this study protects us from that risk.

### Greeting messages

The signal about mood could be skewed by the presence of large amounts of standardised greeting messages in specific seasons, which make use of mood related words, while not denoting the mood of the writer. These standard greeting messages were removed from the data as follows: we ignored any Twitter post containing the word happy, merry, good, lovely, nice, great, or wonderful followed by christmas, halloween, valentine, easter, new year, mothers’ day, fathers’ day, and their variants (e.g starting with a leading # or separated by a dash, a space or ending with ‘s when applicable) was not considered for analysis. We verified that posts matching this pattern were indeed concentrated in very specific days (the expected ones for each holiday).

### Psychometric categories

The words we use to communicate provide an indirect mean of observation to our internal psychological states. Inferring them based on observable samples of behaviour is studied in psychometrics: the word lists in the Linguistic Inquiry and Word Count are well established in this field [1].

They are composed of words and word stems used to measure specific aspects of our psychology. A total of 73 psychometric variables have been defined and associated with a word list. (see S1 Fig—Conditional probability for observing each psychometric variable). These lists were initially assembled and validated by groups of human judges, later iterations in their development relied on statistical methods to reshape them, assure their reliability, and assess their validity in various settings [4]. The psychometric variables attached to the word lists provide indication about the cognitive and emotional states reflected in a sample of text. They also provide general aspects of everyday life such as personal concerns, or the writing style used in each sample.

We refer to a psychological process as any mental path leading to an observable mental state. Amongst the 73 psychometric variables, the distinction is made between those attributed to individual psychological processes, and those designating broader meta-processes. The cognitive processes (*cogproc)* are measured as a whole by one single psychometric variable indicating the expression of the meta-process. They are further declined into individual psychometric variables, each indicating a separate process (*insight*, *cause*, *discrepancies*, *tentativeness*, *certainty*, *differentiation*). Further levels of refinements are made, for example the affective processes (*affect*) include both the positive emotions (*posemo*) and the negative emotions (*negemo)*, with the latter being declined into *anxiety*, *sadness*, and *anger*. Other refinements include *core drives* (*power*, *achievement*, *affiliation*, *focus-risk*, *focus-reward*), *personal concerns* (*work*, *leisure*, *home*, *money*, *religion*, *death*), *social* concerns (*family*, *friends*, *female-referents*, *male-referents*), *time orientation* (*focus-present*, *focus-past*, *focus-future*). The three levels of the complete hierarchy is provided in S1 Table—Processes and meta-processes defined in LIWC 2015.

In this study, we use the categories from all levels, and we refer to them interchangeably as psychometric variables or psychometric categories. Altogether, they provide a wide range of measurements about our internal psychological states, be they cognitive, affective or relative to other clues such as the writing style [1].

### Temporal indicators

From each word list we created a temporal indicator that indicates the hourly expression of the psychometric variable in the population. There are many ways to define such indicators, one way is to count in each hour the number of occurrences of any word in a category and resolve the fraction to the total word volume. The time series of that quantity, the relative frequency of a psychometric variable per hour, is used to investigate temporal changes in the population.

The temporal indicators we use are formed by computing the frequency of individual words in a robust way, first improving each time point estimate by smoothing it with the corresponding estimates at seven days distance, preceding and following to ensure we do not introduce artifacts in hourly comparisons through the week while still keeping the hourly differences between corrected estimates. Then, the relative frequency is resolved for each word in every hourly sample, by expressing the frequency of a word in a sample over the total volume of the top 100K most frequent words.

Our temporal indicators are obtained by averaging the signal of all individual words denoting a psychometric variable, yielding a time series of time point averages across a word list. Since the frequency of words in the natural language is determined by the Zipf’s law [13] two problems arise when averaging the signals. A simple sum of frequencies for all words in the list would only reflect changes in the top most frequent words, and the long tail of low-frequency words would be affected by a high estimation error.

We address the first issue by standardising the time series of a word within the 24-h day so each single day receives zero mean and unit standard deviation before averaging the indicator. In this way each word contributes equally towards the score of a category. In particular if two words with different baseline volume show an increase of 100% at a specific time, our indicators can experience an increase of 100% at that time. This strategy also removes the effect of long trends in individual word volume (e.g it is known that baseline volume of mood words changes seasonally on Twitter [9,10]), and ensures that only variations that happened within the 24-h cycle are reflected in the temporal indicators. We acknowledge the standardisation procedure can amplify the second issue linked with the Zipf’s law. We addressed this by discarding the 50% least frequent words in each list, after observing that the 50% most frequent words accounted for over 98% of all occurrences in each of the 73 categories.

As a sanity check, we also computed indicators that account for the natural volume in each individual word, and we compared with our own indicators (see Results –Dynamics of the Diurnal Rhythm). Because the baseline volume of words grows exponentially with their rank frequency in a corpus of text, the two methods are not equivalent. Our standardisation strategy ensures that in this uncontrolled population finer structures in the indicators account for discrepancies in word usage [14], and do not only account for the fluctuations of the top most frequent words.

### Diurnal Variation Profile

**Fig 1:**
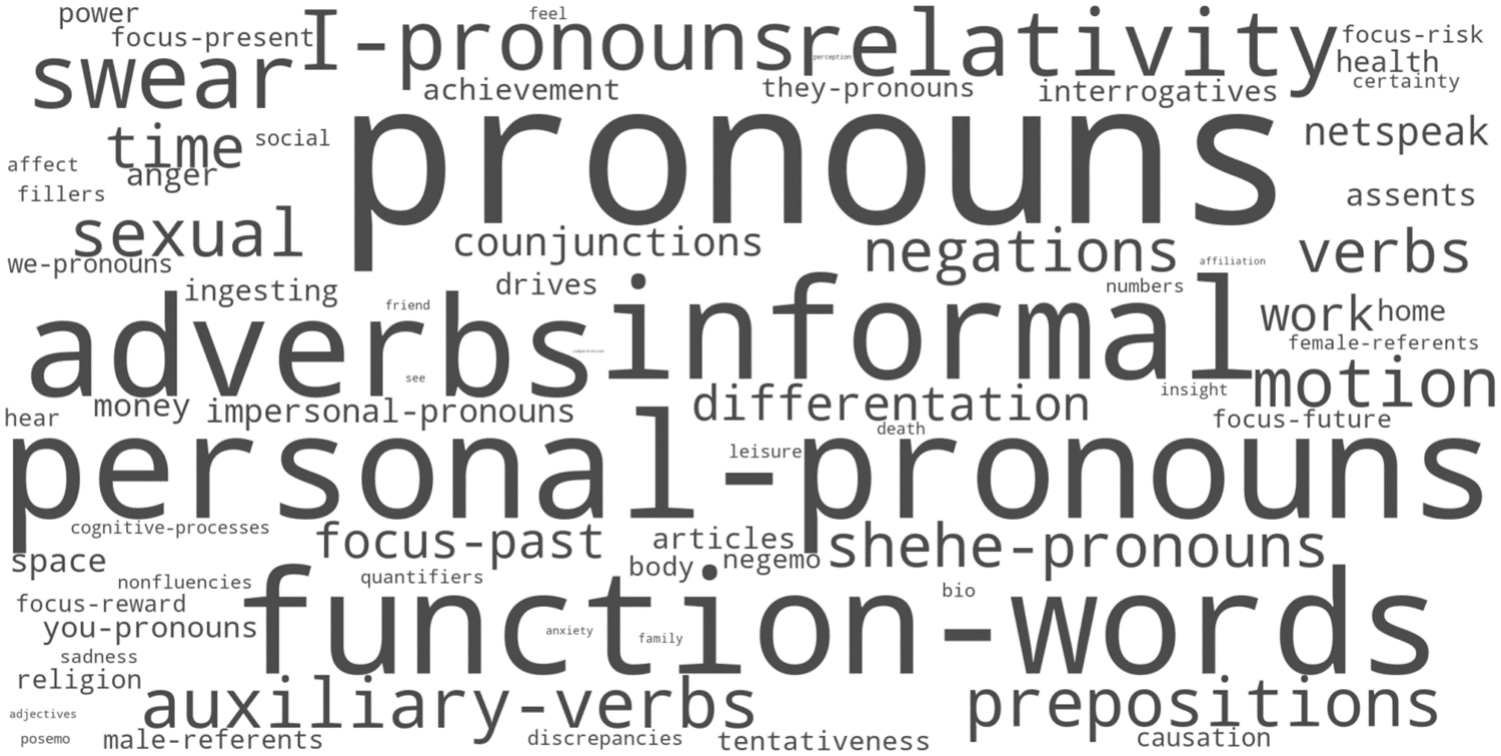
Levels of periodicity in the temporal expression of the 73 psychometric variables at period 24-h. The label of a category has size proportional to the percentage of variance explained in the four years fluctuations of the indicator by the profile of diurnal variation.

Each temporal indicator is summarised within the 24-h cycle by a Diurnal Variation Profile (DVP), defined as the 24-bin series of time point averages across each word and each day in the period under study. The DVP is characterised as a single period of the periodic times series (with period 24-h) that minimizes the residual sum of square with the temporal indicator. In that sense the squared correlation between the DVP and the temporal indicator is interpreted as the percentage of the variance in the indicator that is explained by the DVP (see Fig 1). In this study the DVP was computed separately for the weekends and for the weekdays, it was then averaged, and standardised for convenience.

## Results

In this section we present statistical results showing that the temporal indicators of the 73 psychometric variables are indeed periodic, most of them having 24-h as their dominant period. Then we consider their diurnal pattern of change, finding that 85% of the variance across them can be explained by two independent factors, studied in detail. The DVP of these two factors is robust across the different days of the week, we discuss how they correlate with emotions, then with cognitive states and changes in writing style.

### Dominant cycles

By Fourier analysis, we see that all the 73 time series have strong periodic structure, and all have a 24h component, which for 65 of them is also the dominant component. This step is performed by first standardising each individual word time-series across the four years, (instead of each single day as done later, see Materials and methods –Temporal Indicators), to keep longer term fluctuations in the signal. The 24-h Fourier component appears as the largest periodic oscillation with period under a year in 65 temporal indicators out of 73. The second largest dominant period corresponds to a weekly cycle, over-expressed in the working days (*cognitive-processes*, *insight*, *comparatives*), or in the weekends (*family*). A 12-h dominant cycle appears in both *focus-future* and in the positive emotions (*posemo*), with the latter discussed in [3]. The category *affiliation* shows an 8-h dominant cycle followed by a dominant weekly cycle, and the category *perception* a 4-month dominant cycle followed by a 24-h dominant cycle.

### Diurnal Variation Profiles

**Fig 2:**
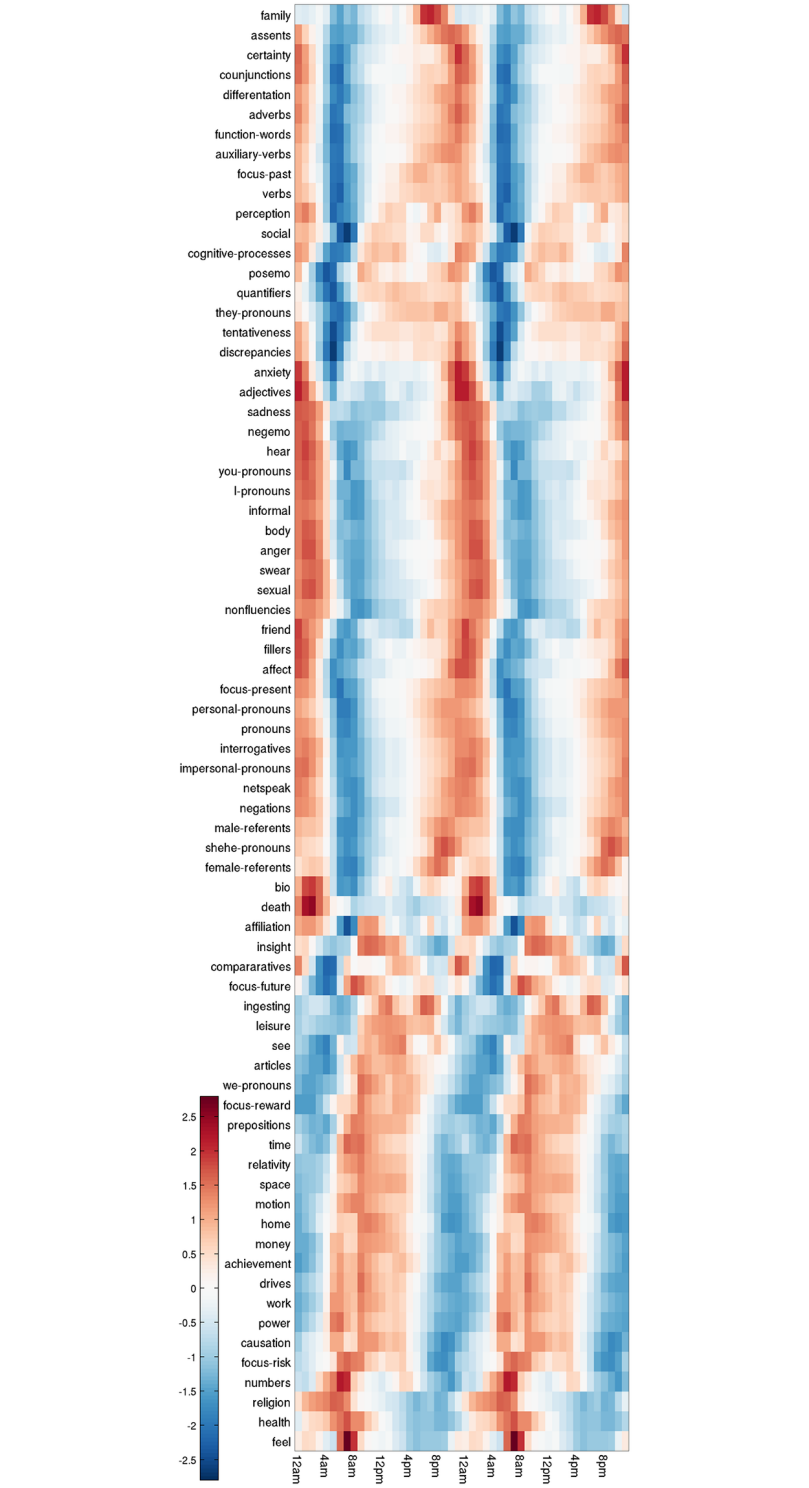
Profiles of diurnal variation of the 73 psychometrics variables.

We summarise these diurnal movements by extracting a Diurnal Variation Profile (DVP) showing the variations of a psychometric variable during the day (see Material and methods - Diurnal Variation Profile). The DVP was found significant at 1% level in all 73 cases by comparing the levels of variance explained in the four years series to the levels obtained in 100,000 random permutations. Fig 1 compares the levels of periodicity in each case: the size of the labels is proportional to the percentage of the variance explained by the DVP. The diurnal structure was the most pronounced in the pronouns, suggesting important changes in writing style (a detailed discussion is found in Results –Cognitive states and CDI index). It was the least pronounced in the *comparatives*, examples of which include "farther", "smaller", or "longer". All 73 profiles of variations are made available in Fig 2, which shows the 73 DVPs sorted in a way to emphasize their correlation. Rows represent in shades of red (resp. blue) values above (resp. below) the average 24h value. The figure shows that most indicators tend to cluster into few broad categories, many indicators being temporally correlated to each other.

We investigate the underlying structure of these 24-h movements using a type of Factor Analysis, that consists in extracting the orthogonal directions accounting for the maximum of the variance between the profiles. Note that the factors extracted by Principal Components Analysis (PCA) [15] are unique up to their sign, therefore when interpreting the results there is no reason a priori to prefer a factor to its opposite. As a convention, we choose the sign that gives positive correlation between a factor and its most correlated DVP. This observation will be important in the final discussion, when possible neuroscience correlates of these factors are discussed: since high levels of expression of a factor at a given time could also be interpreted as low levels of expression of its opposite.

**Fig 3:**
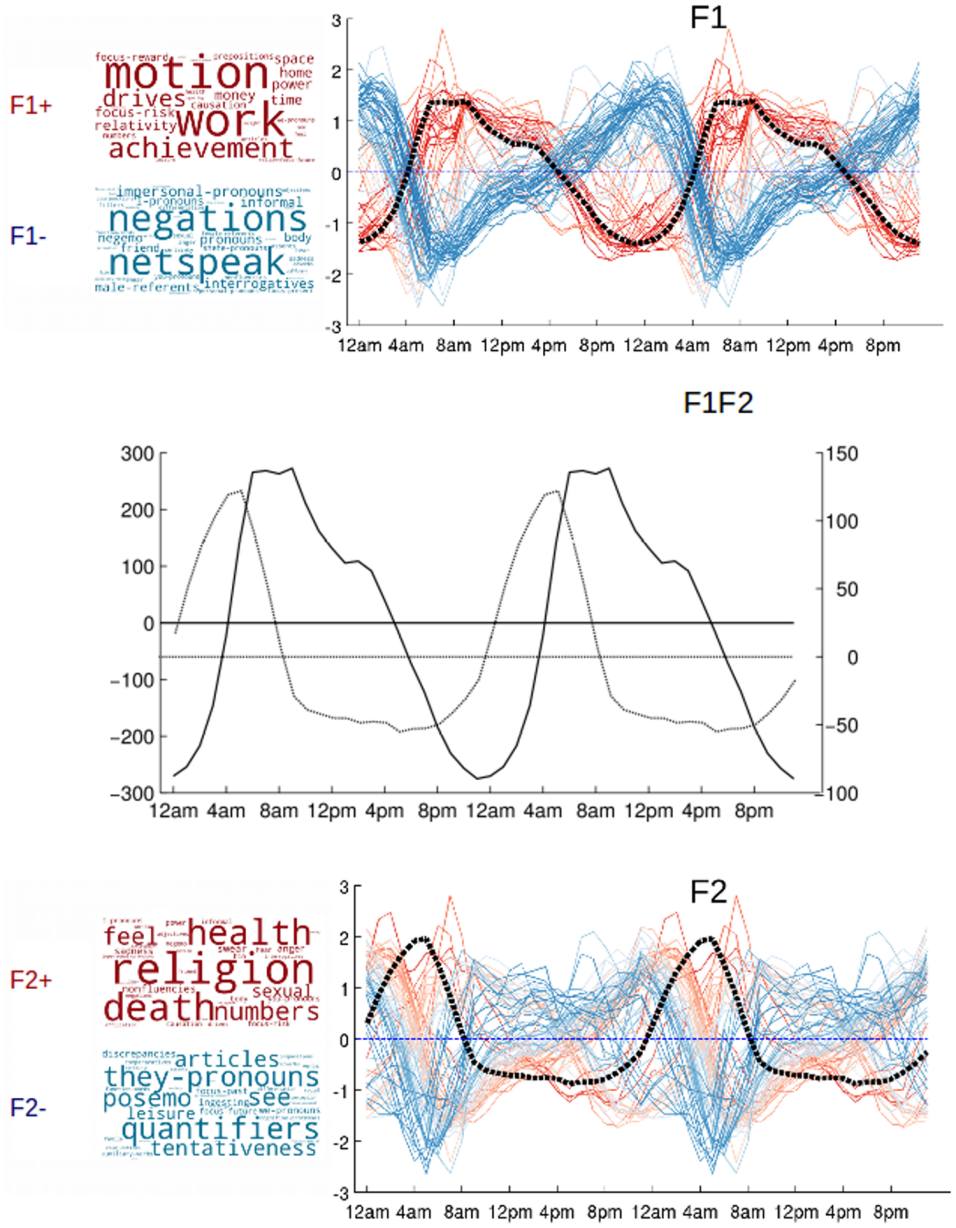
Leading factors behind the Diurnal Variation Profile of the 73 psychometric categories. The first leading factor (F1) and the second leading factor (F2) are shown separately. The profiles that contribute positively to the factors are plotted in red and displayed in the box above the x-axis (F1+ and F2+). The profiles that contribute negatively are plotted in blue and displayed in the box below the x-axis (F1- and F2-). The label of a profile has size proportional to the magnitude of its loading on the factor. The two leading factors are plotted in their respective scales for comparison (F1F2). The x-axis indicates the hour.

Our analyses reveal two factors, shown on Fig 3, that explain 85% of the variance between the profiles. The first leading factor (F1) peaks in the morning with maximal expression between 6am and 10am (see Fig 3 - F1), and accounts for 64.8% of the variance between the 73 profiles. The second leading factor (F2) peaks in the late night with maximal expression between 3am and 5am (see Fig 3 - F2), and explains 20.6% of the variation across profiles. (*) Our results indicate the time between 3am and 10am as an important moment of changes in our mental states (see Fig 3 - F1F2).

The factors identified also hold when accounting for the natural volume in each individual word (see Materials and methods –Temporal Indicators), as indicated by the comparison with our indicators of diurnal variations (F1 ρ = 0.98; P = 2.70e-16; N = 24; and F2 ρ = 0.98; P = 5.48e-16; N = 24). Our standardisation strategy ensures that finer structure in the 73 DVPs (see Figs 2 and 3) are not dominated by the top most frequent words in this uncontrolled population.

**Fig 4:**
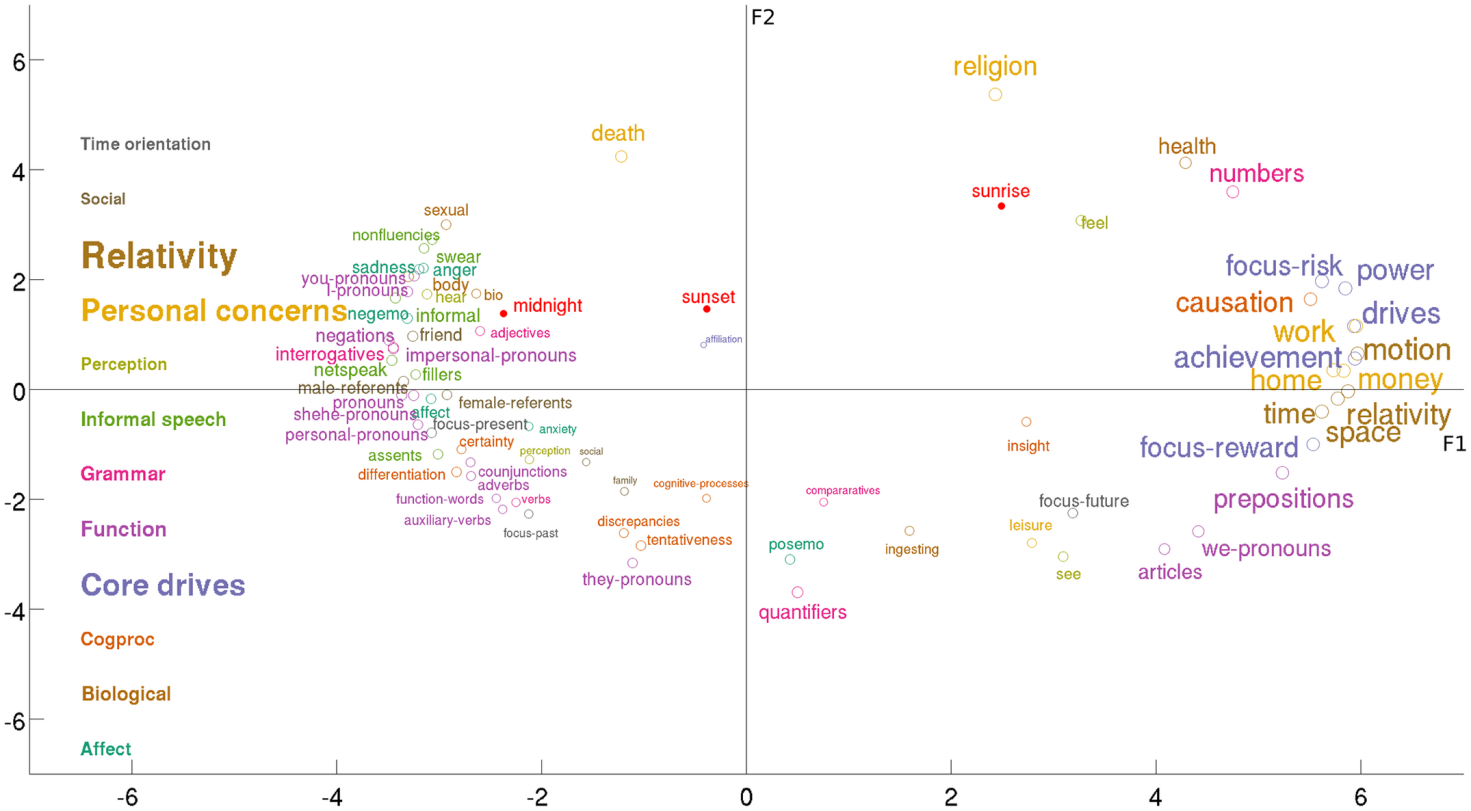
Scatter of the 73 psychometric variables against the two orthogonal factors. The profile of variation of each variable is mapped to the embedding spanned by the two leading factors, F1 (x-axis) and F2 (y-axis). The size of a label is proportional to the magnitude of the largest loading score in the embedding. We have listed the broader meta-processes on the left of the image with different colours, and size relative to the average size of the individual variables that compose them. Marker words (sunrise, sunset, midnight) which are known to peak at specific times were added to guide reading.

The factors, obtained as the orthogonal directions that account for the maximum of the variance across profiles, are used to scatter the 73 psychometric variables on these two directions (see Fig 4).

The variables that associate to the greatest extent with the first leading factor (F1) are the *core drives* (*achievement*, *power*, *focus-reward*, *focus-risk*), *personal concerns* (*work*, *home*, *money*), *relativity* (*motion*, *space*, *time*), and *causations* in the cognitive processes. Important changes in writing style are associated with the expression of this factor: *articles*, *prepositions*, and first person plural pronouns (we-pronouns) (see discussion in Results –Cognitive states and CDI index).

The second leading factor (F2) is maximally expressed in the late night, the most prevalent category on the social platform before the time of peak is *death*, this time corresponds with low positive emotions (see Figs 2 and 4), it then shifts towards *religion* as the night transitions towards the early morning, and as F2 stops being expressed in the population.

**Fig 5:**
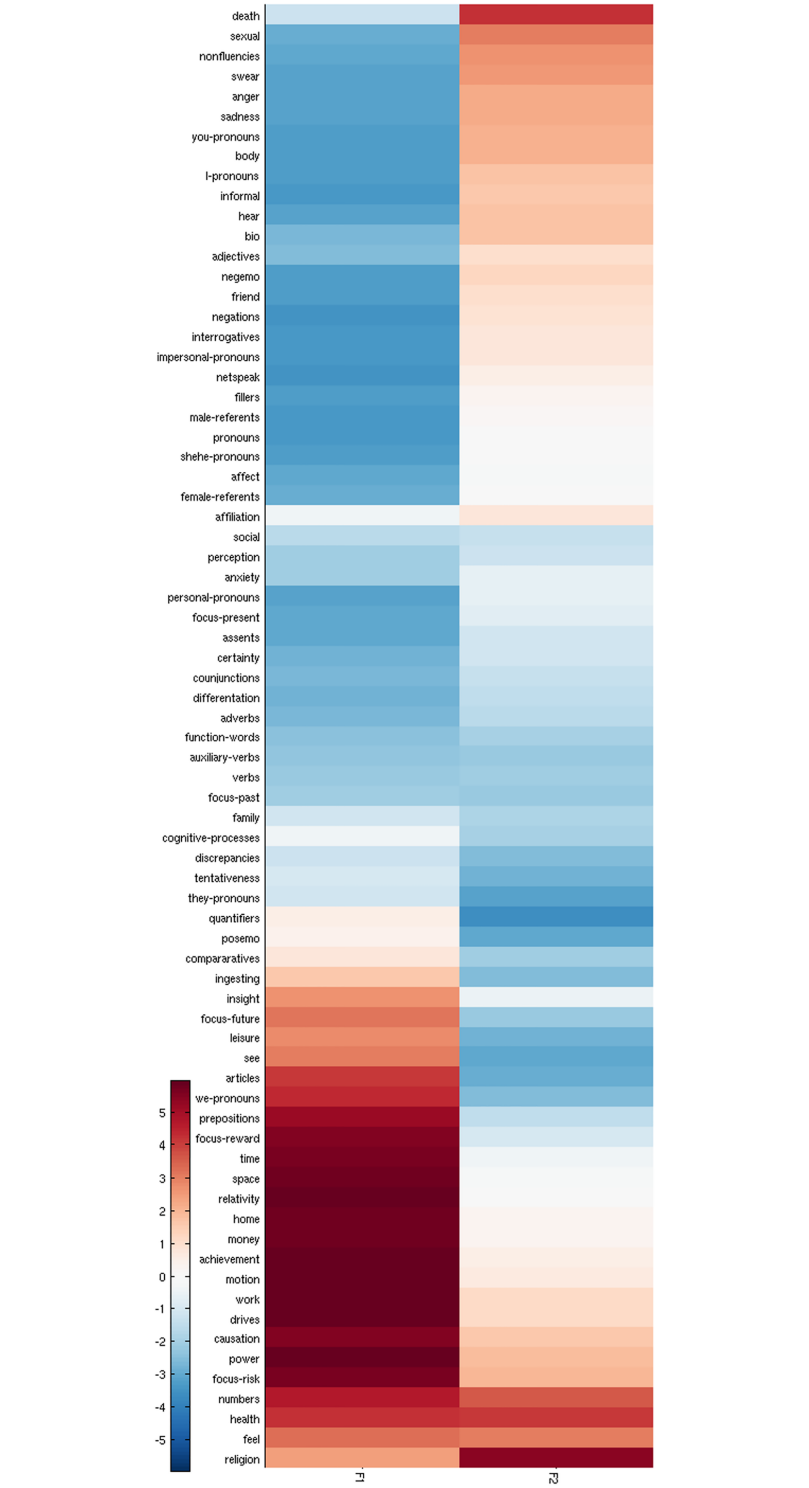
Mappings of the two types of thinking identified against the 73 psychometric variables. The colours indicate the loading scores of the 73 categories onto F1 and F2.

The factors reflect two independent types of thinking identified on the social platform. We refer to the former as categorical thinking (F1), and the latter as existential thinking (F2). We provide their complete mapping along the 73 psychometric variables in Fig 5. The 73 profiles are arranged in a heatmap where they are displayed in rows sorted by pairwise distance (partial order) in the embedding spanned by the two leading factors. The columns store the loading scores of each category along the corresponding factor: shades of red indicate positive association between a category and the factor, and shades of blue indicate negative association. Finer groups of co-expressed categories emerge from the heatmap, with red blocks indicating over-expression of the corresponding factor, and blue blocks indicating under-expression of the factor.

The group of variables characteristic of categorical thinking or over-expressed F1 emerge at the bottom of the heatmap (strong red on the first column). They are further decomposed into two groups of variables. The first (*causation*, *power*, *focus-risk)* activates when existential thinking (F2) is still expressed (red on the second column). The second (*articles* suggesting the usage of concrete nouns, *prepositions* relative to categorical thinking [5], and the first-person plural *we-pronouns)* activates when existential thinking is no longer expressed on the social platform (blue on the second column).

Existential thinking, characterised by high usage of *death* and *religion (*strong red on the second column), is further specified by reduced expression of *quantifiers* (strong blue on the second column), examples of which are “few”, “many”, “much” [1].

At the crossing of the two types of thinking: the population tend to *hear* in the early night, while they tend to *see* during daytime (see Figs 2 and 5). The early night is also marked with expressed negative emotions (negemo) and *informal* language before it turns to existential thinking (see Fig 2). The daytime is marked with *insight*, *focus-future*, and *leisure* after it turned to categorical thinking.

### Stability across days of week

**Fig 6:**
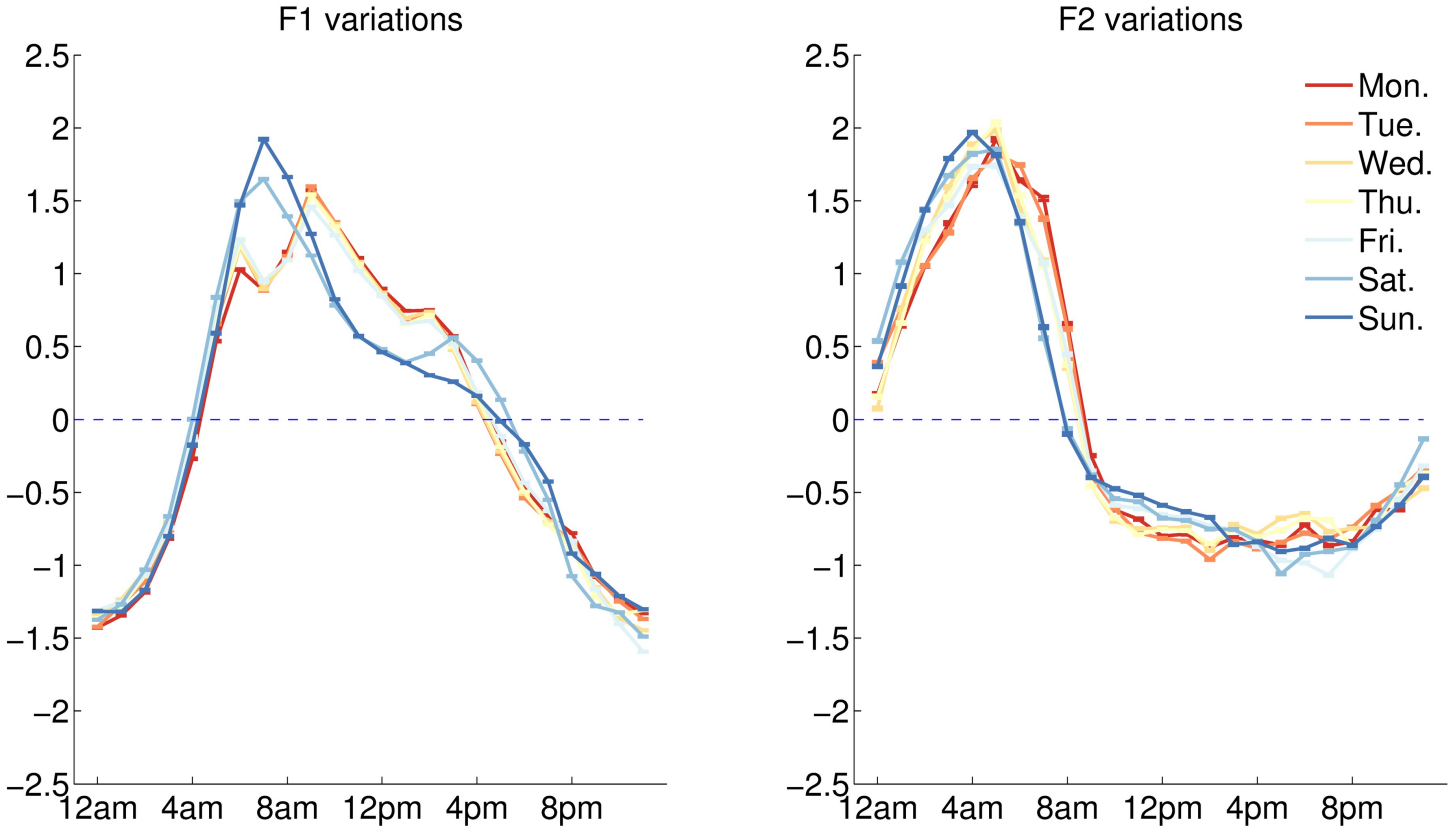
Diurnal variations of the first leading factor (left) and second leading factor (right) by each day of week. The x-axis indicates the hour.

We computed the DVP of the two latent factors for different days of the week, as a way to check if these factors are affected by the change in routine that are associated to different days. We find that the overall behaviour of the two factors is stable across the week, although some smaller differences can be seen between weekends and weekdays. (see Fig 6). To enable fine comparisons, we compute a bootstrap estimate of the 95% Confidence Interval (N = 100 samples) for the reported quantity, and we provide both the bootstrap mean and the 95% CI. In each case we report the variations of the factor obtained by standardising the temporal pattern in the 24-h interval.

**Fig 7:**
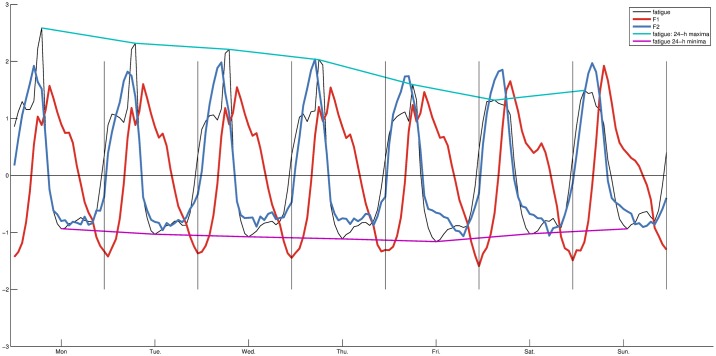
Comparison between variations of F1, F2, and weekly levels of fatigue.

On the figure, F1 is seen to react adversely to an event occurring in the morning of the worked days. F2 does not show the same behaviour but stops being expressed progressively earlier in the morning. To give context to these observations we compare these patterns with weekly levels of fatigue [3]. On Fig 7, we notice exceptional levels during peak expression time of F1. The effect is degressive through the week, and fits with the progressive behaviour of F2 in the morning. In the weekend, fatigue does not peak in the morning, and the lower mood of the worked days occurring during the morning rush hour is replaced by a feel-good time (see Results –Emotional states) marked with relatively higher F1 and lower F2.

Extracting separate factors for the weekend when the population is not engaged with the weekday work schedule, in contrast for the weekdays when they are, confirms the strong agreement between the two periods, with both the first leading factor (ρ = 0.95; P = 1.27e-12; N = 24) and the second leading factor (ρ = 0.97; P = 1.93e-15; N = 24) showing robust consistencies in response to the change of activity. We provide the detailed mappings for each day of week in S2 and S3 Figs.

### Emotional states

In this section we are interested in interpreting F1 and F2 by how they interact with emotional states. In the next section we interpret F1 in relation with the cognitive states and the writing style.

**Fig 8:**
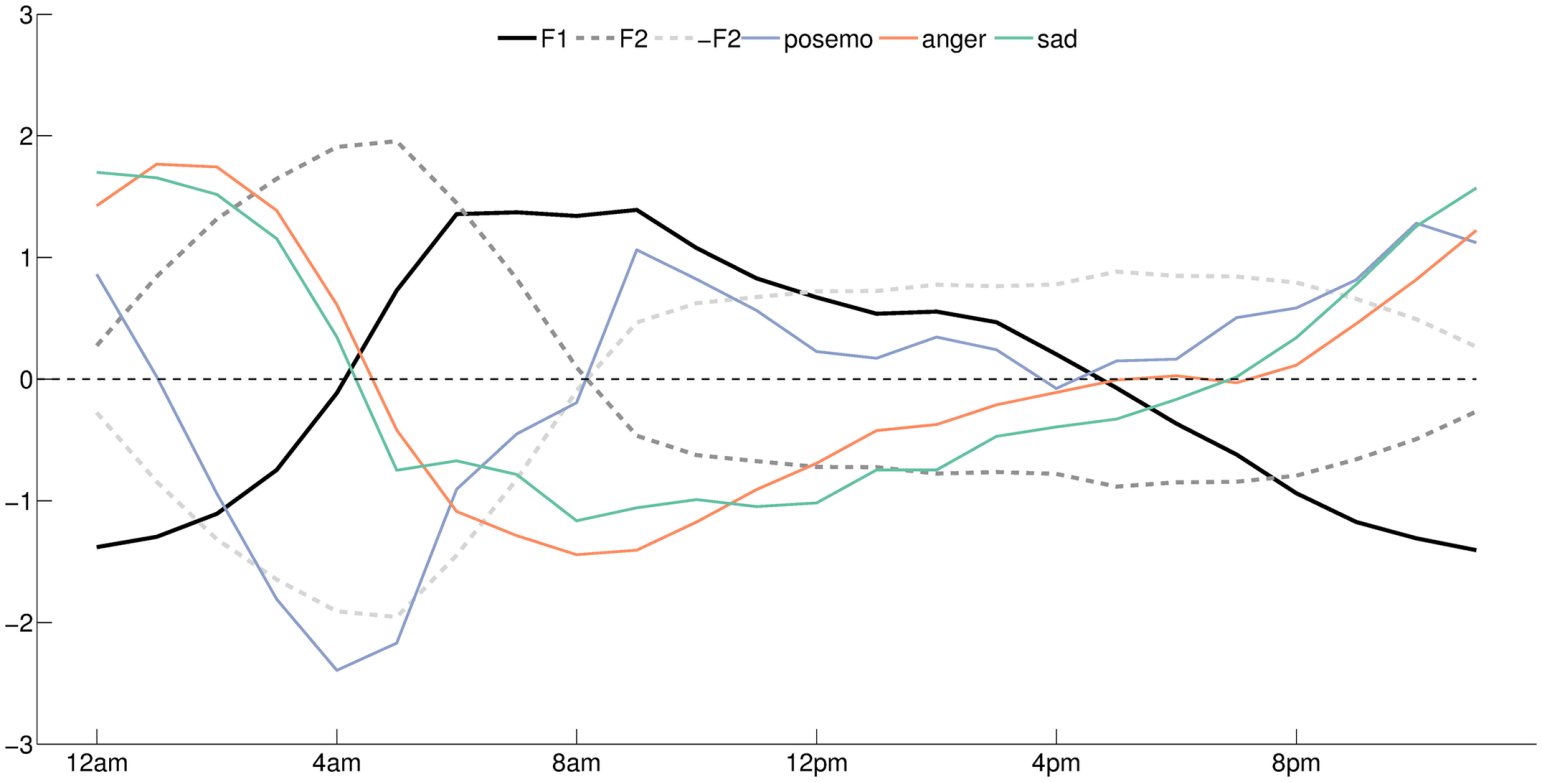
Comparison between the variations of posemo, anger, sadness, and the two leading factors. The x-axis indicates the hour.

A strong association between the circadian rhythm and emotions has been reported under various conditions in the literature [16]. In the context of the present study, F2 shows a pattern that anticorrelates with the positive emotions. F1 shows a pattern that anticorrelates with negative affect (see Fig 8).

To detail these effects, we provide the profile of variations of the mood indicators analysed in [3] (*posemo*, *anger*, and *sadness)* and we compare with the variations of the two factors by each day of week (see S4 Fig. Days of week comparison between the variations of posemo, anger, sadness, and the two leading factors.). In each case we observe stable profiles of variations with smaller deviations occurring in the morning.

The population wake up in the best mood on Sunday with high positive emotions and low negative emotions (*anger*, and *sadness*) expressed after 6am. In the couple of hours that follows, the working days are instead associated with relatively low mood characterized by low positive emotions and increased *sadness*. These differences occur during the morning rush hour marked with degressive levels of fatigue through the week (see Fig 7).

As noted in [3], the profiles of *anger* remain more stable. In the context of the present study *sadness* anticorrelates with F1 and shows differences during the morning rush hour of the worked days when the population express exceptional levels of fatigue. This is also the case for the positive emotions that anticorrelate with F2, their morning onset interacting with peak expression of F1. These observations are only intended to aid with interpretation of F1 and F2, and not to suggest causation.

### Cognitive states and CDI index

The consistent observation of diurnal variation in the usage of affective words on the social platform [3,8] is also found to manifest via the writing style and the cognitive variables in our data. The function words (e.g articles, prepositions, pronouns), indicative of writing style, have been related to a core dimension of thinking style called the, Categorical Dynamical Index (CDI) introduced in [5], and found to be an indicator of two different modes of thinking: categorical and dynamical. The function words are found to express at a high periodic rate on the social platform (see Fig 1), in strong association with F1. They are used in the language to connect words together and as such they reflect writing style [1]. While the articles and the prepositions, indicative of categorical thinking [5], are strongly correlated with the factor, the high usage of the pronouns suggests a more dynamic type of thinking indicative of narrative discourse, they are maximally expressed in the evenings and nights and strongly anticorrelated with F1. The first person plural pronouns (*we-pronouns*) stand in opposition of phase with all other pronouns, they are expressed during the daytime, showing association with the factor.

**Fig 9:**
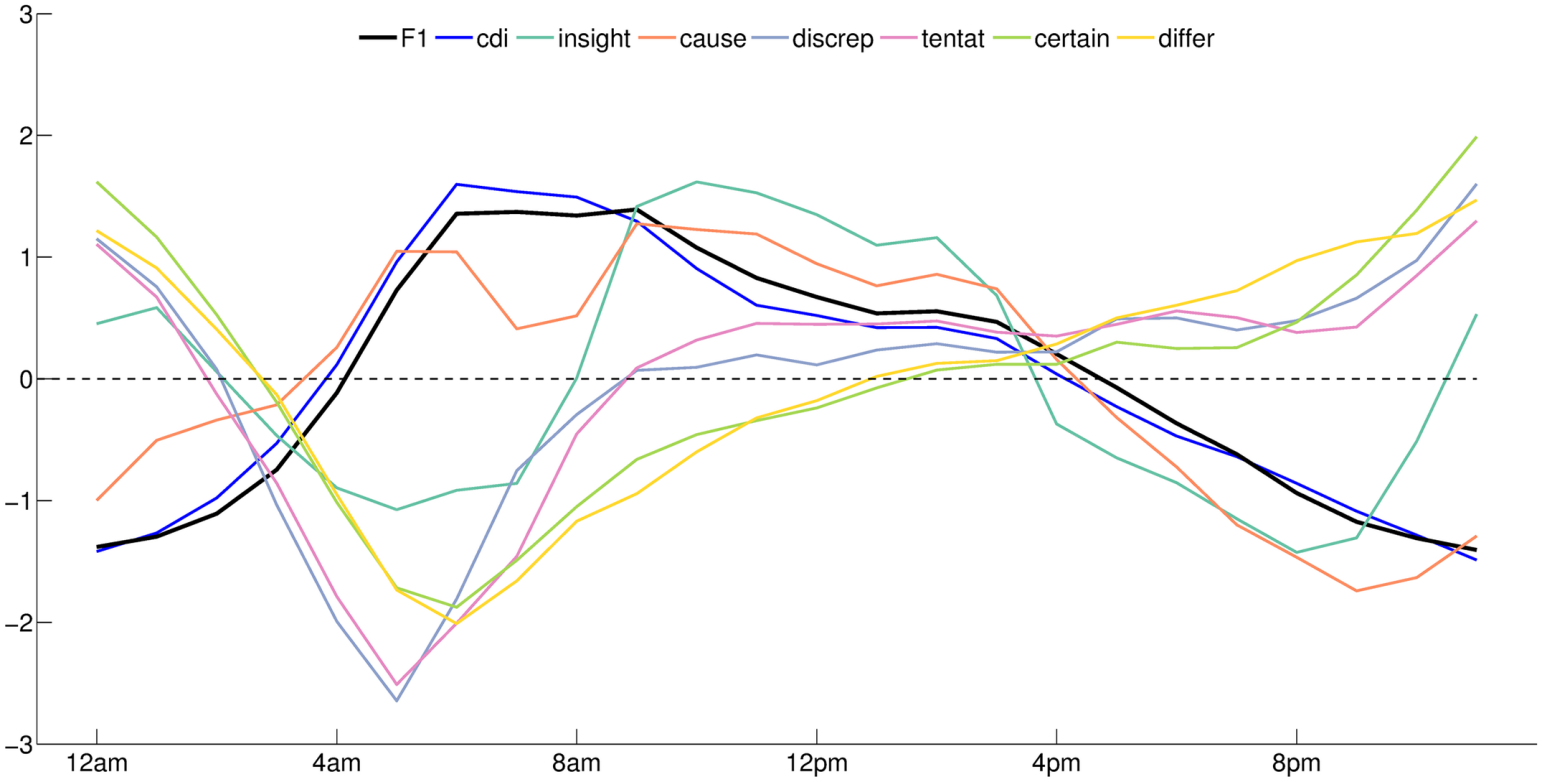
Comparison between the variations of cognitive activity, writing style, and the first leading factor. The x-axis indicates the hour.

We computed the DVP of the CDI [5], that indicates the expression of *articles* or *prepositions* in the absence of *pronouns*, *conjunctions*, *adverbs*, *negations*, and *auxiliary verbs* (see Fig 4). Comparing with the variation of F1, we found a strong association between the two quantities (ρ = 0.99; P = 1.57e-19; N = 24). A manual screening of the tweets posted during peak expression time of the CDI index and F1 reveals a tendency to share updates, information, news stories, or links in general. The association between categorical thinking and high F1 is completed by high expression of causations, indicative of problem solving and logical thinking in the mornings (see Fig 9). All other cognitive variables, but *insight*, are found to anticorrelate with F1, with high expression in the evenings and night, suggesting a tendency in the population to express *discrepancies*, *tentativeness*, *certainty*, and *differentiation* when the factor stops being expressed.

The category *insight* shows a dominant weekly cycle, it is expressed during the day, on the weekend it is also expressed late in the night (see S5 Fig. Days of week comparison between the variations of cognitive activity, writing style, and the first leading factor). These patterns are found to be stable in each day of week, smaller differences are observed during the morning rush hour marked with degressive levels of fatigue through the week. As for the case of emotional states, these observations are only intended to aid with interpretation of F1 and F2, and not to suggest causation.

## Discussion

In the present study we have established that a variety of psychometric indicators extracted and averaged from Twitter present a strong diurnal pattern across a large population. These results show that we use different languages at different times of the day, thus revealing different concerns in our daily routines, and variation in our underlying psychological processes. We have also established that 85% of their variations is explained by only two factors, the first peaking between 6am and 10am, and the second peaking between 3am and 5am. These patterns are found to be consistent in the general population between periods of work, and periods of rest. We have also observed that F1 correlates with changes in writing style and a psychometric dimension that separates categorical thinking from dynamical thinking (CDI); and F2 anticorrelates with positive emotion. This study is designed to reduce inter-individual variation by averaging over a large population.

While these trends might not necessarily be due only to physiology, and other factors can be at play, it is nevertheless interesting to examine these facts alongside known physiological facts.

Circadian expression of genes regulates many physiological processes including sleep/activity cycles, body temperature and metabolism. Although the suprachiasmatic nucleus in the hypothalamus is the master clock coordinating many of these activities it is becoming increasingly clear that slave oscillators in other brain regions coordinate many aspects of sensory, motor and psychological function including memory formation [17]. Fear memory is also circadian and interestingly the amygdala shows clear patterns of circadian clock gene expression, which can be modified by glucocorticoids [18]. Major brainstem activating circuits also show circadian regulation with serotonin synthesis and firing of serotonergic neurons maximal in the inactive state [19] while acetyl choline in the hippocampus is higher during the active phase [20]. In addition to these changes in activity of neural circuits there is the circadian activity of the glucocorticoid hormones that markedly affect many neural circuits, and of melatonin- an essential part of the sleep wake system [21, 22, 23].

It is therefore not surprising that aspects of psychometric activity are also sensitive to circadian processes. The present study is not able to define a causal relationships between these factors, but F1 occurs at the time when serotonin activity will be falling and cortisol rapidly rising and indeed the characteristic variations of Cortisol production are remarkably similar to the diurnal variation of F1, marked by a rapid increase from 3am to early morning, reaching maximum levels between 6am and 10am, then slowly decreasing until the rest of the day [24] (see Fig 2). On the other hand, F2 occurs at maximum serotonin activity and minimal cortisol.

These results show a prevalence for categorical thinking at times of increasing midbrain raphe serotonin synthesis and heightened cortisol production with a prevalence for dynamical thinking at times of low cortisol production and increasing melatonin production. Whether these associations have any implications for the role of serotonin and cortisol in the categorical and existential thinking would be pure conjecture but there is great scope for further investigation into the mechanisms underlying our data, and how it may change during disease states. Future work will consist in separating all these effects.

## Supporting information

S1 Table Processes and meta-processes defined in LIWC 2015. (XLSX)

S1 Fig Conditional probability for observing each psychometric variable. (TIF)

S2 Fig Days of week mappings of the first leading factor—Categorical thinking (F1) against the 73 psychometric variables. (TIF)

S3 Fig Days of week mappings of the second leading factor—Existential thinking (F2) against the 73 psychometric variables. (TIF)

S4 Fig Days of week comparison between the variations of posemo, anger, sadness, and the two leading factors. (TIF)

S5 Fig Days of week comparison between the variations of cognitive activity, writing style, and the first leading factor. (TIF)
