# Supplementary figures and images for: Diurnal variations of psychometric indicators in Twitter content
Source: PLoS One. 2018 Jun 20;13(6):e0197002. doi: 10.1371/journal.pone.0197002 (PMC6010242; doi:10.1371/journal.pone.0197002)

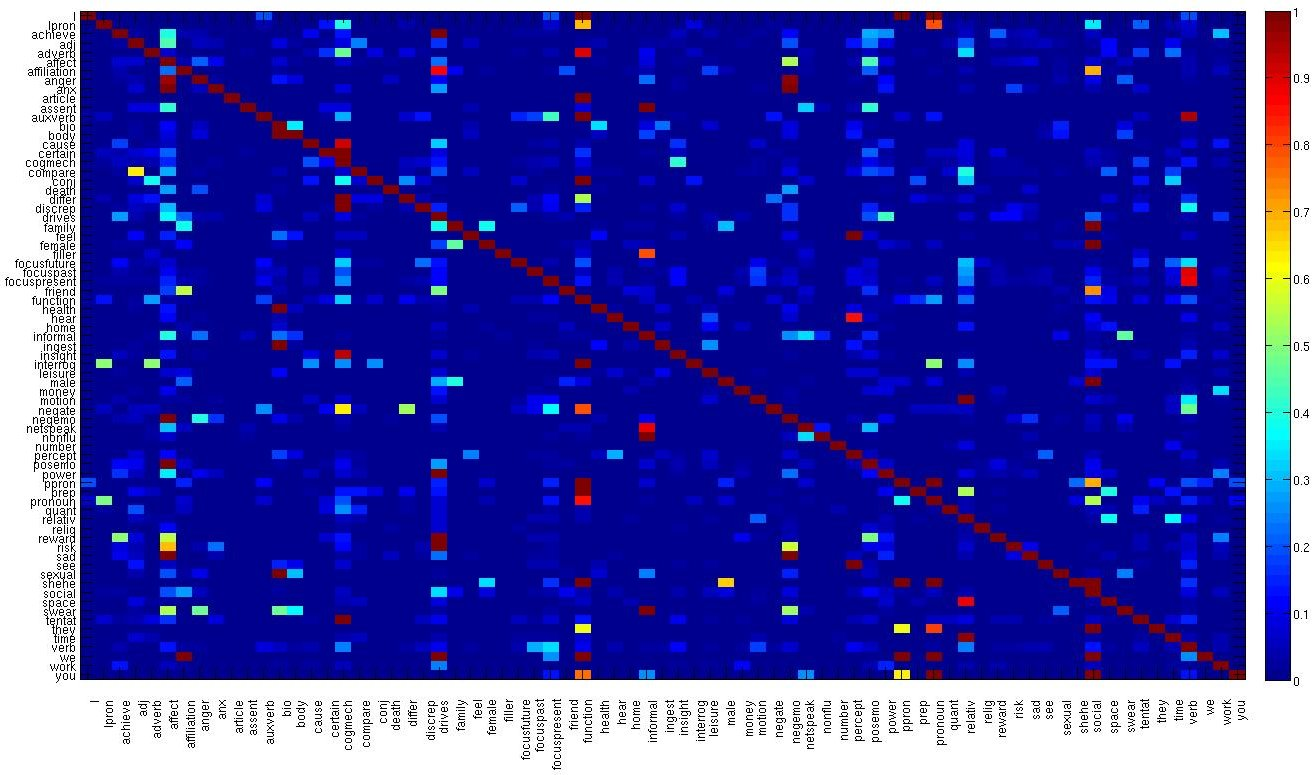

Supplement: S1 Fig — (TIF) [file pone.0197002.s002.tif]

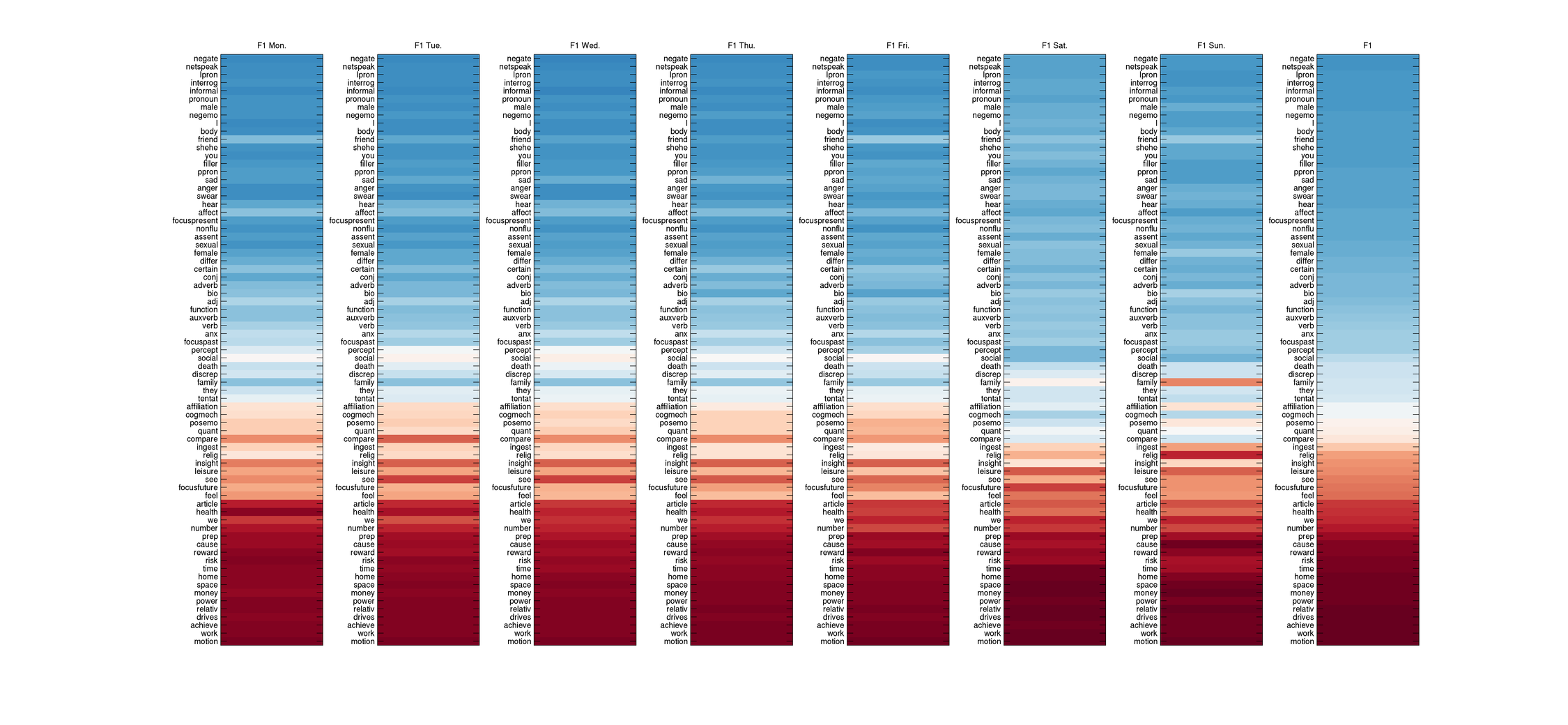

Supplement: S2 Fig — (TIF) [file pone.0197002.s003.tif]

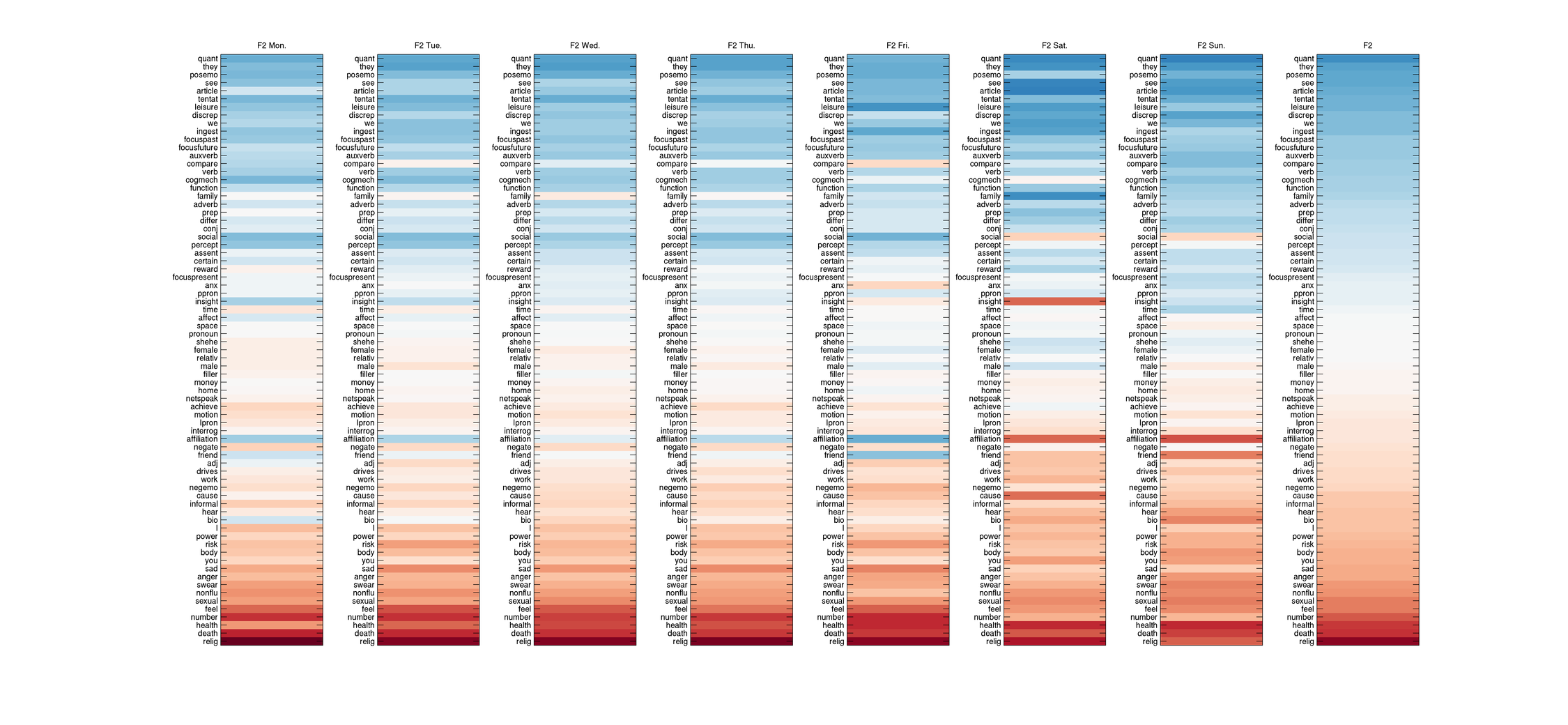

Supplement: S3 Fig — (TIF) [file pone.0197002.s004.tif]

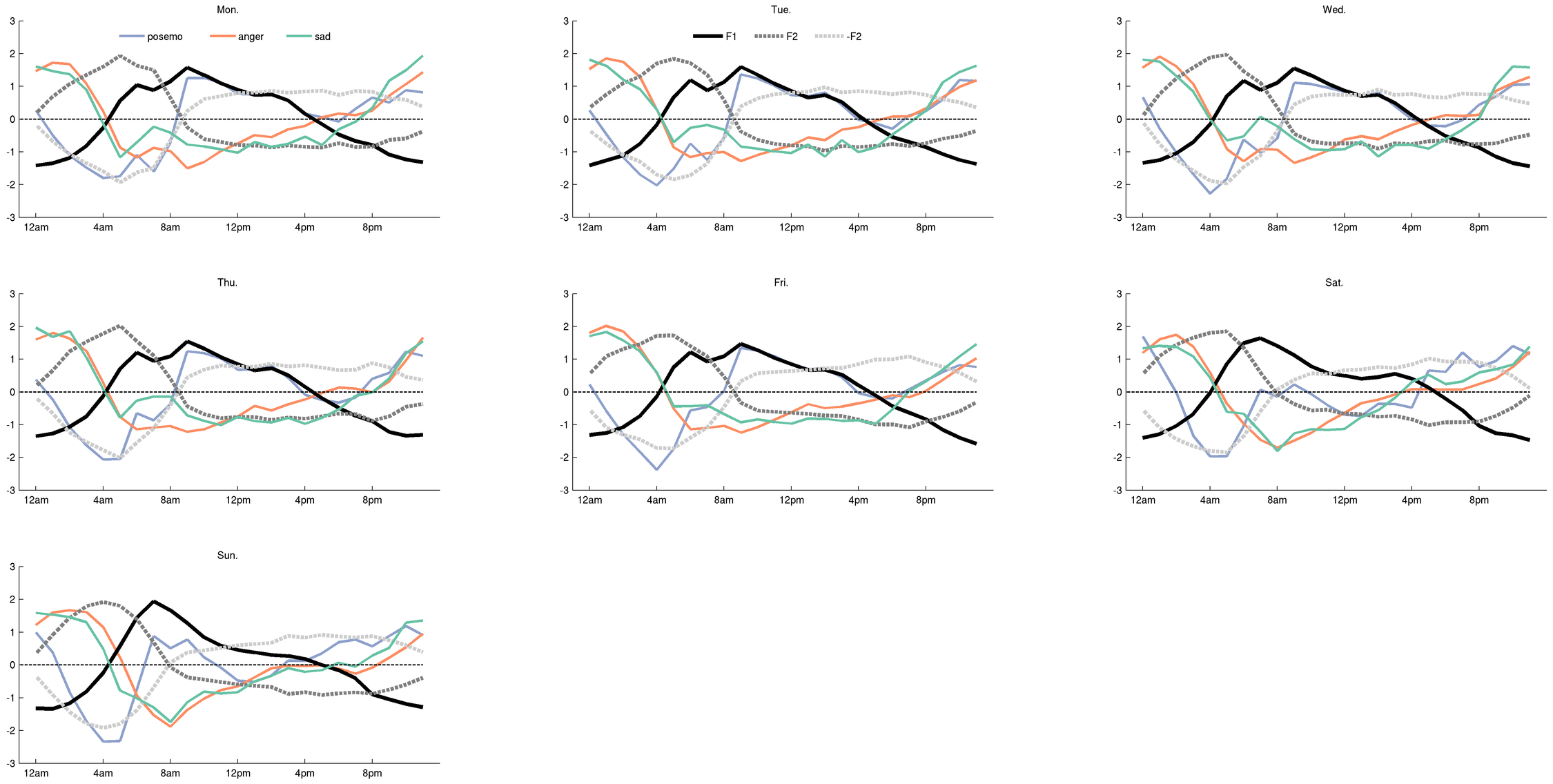

Supplement: S4 Fig — (TIF) [file pone.0197002.s005.tif]

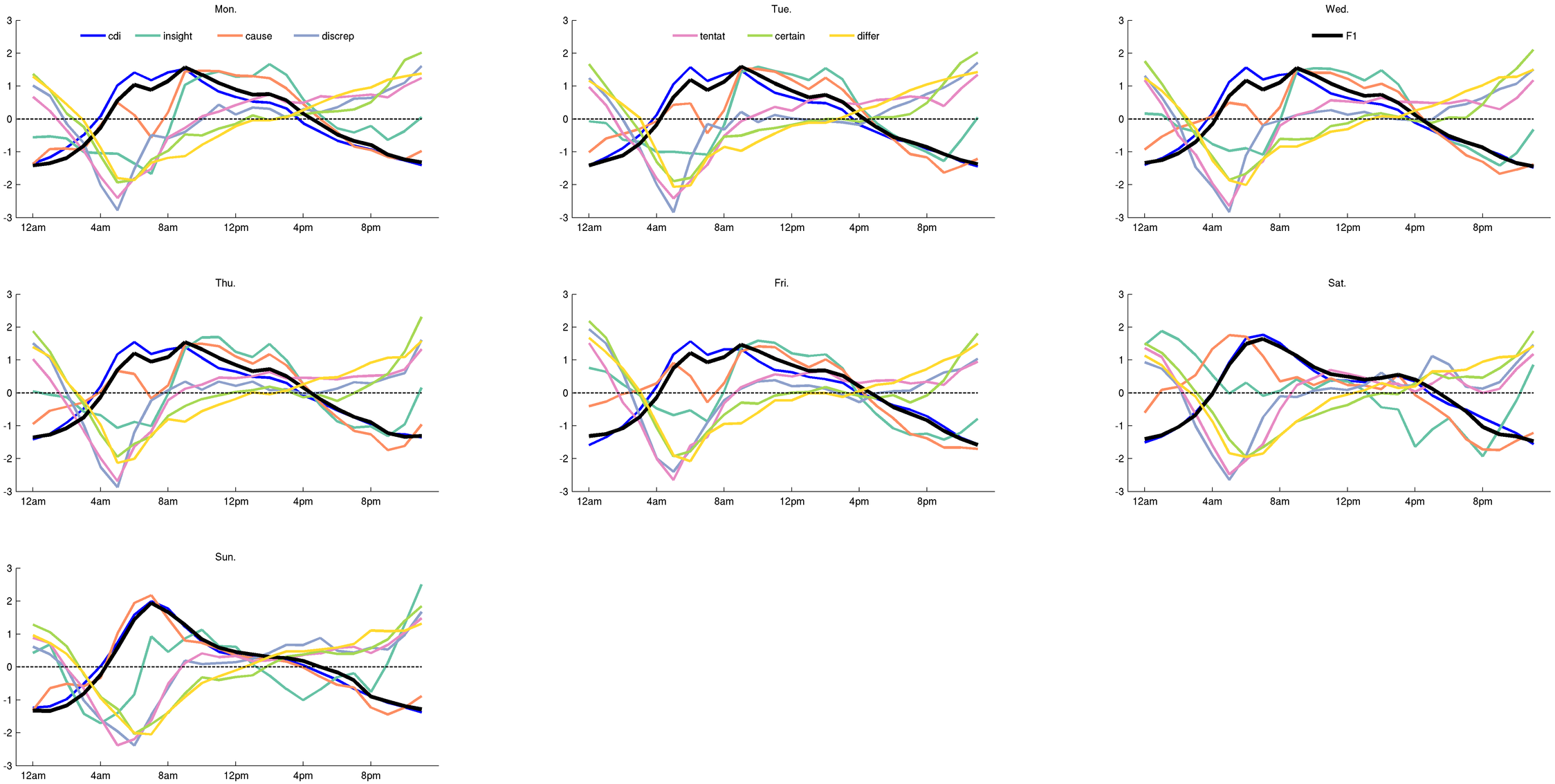

Supplement: S5 Fig — (TIF) [file pone.0197002.s006.tif]
